# Effect of Distance and Duration of Illumination on Retinal Ganglion Cells Exposed to Varying Concentrations of Brilliant Blue Green

**DOI:** 10.14740/jocmr2085e

**Published:** 2015-05-08

**Authors:** Kakarla V. Chalam, Wenhua Li, Keyvan Koushan, Sandeep Grover, Sankarathi Balaiya

**Affiliations:** a Department of Ophthalmology, University of Florida College of Medicine, Jacksonville, FL, USA

**Keywords:** Brilliant blue green, Retinal ganglion cells, Chromovitrectomy, Endoillumination, Cytotoxicity

## Abstract

**Background:**

The objective of the study was to determine the safety parameters of using brilliant blue green (BBG) for chromovitrectomy by assessing the cytotoxicity of BBG on cultured retinal ganglion cells (RGCs) exposed to illumination.

**Methods:**

RGCs were exposed to two concentrations of BBG (0.25 and 0.5 mg/mL) under metal halide illumination at varying distances (1 and 2.5 cm), intensities (990 and 2,000 Fc), and durations (1, 5 and 15 minutes). Cell viability was assessed using the WST-1 and CellTiter 96^®^ AQueous One solution cell proliferation assays.

**Results:**

Using the WST-1 assay, with high-intensity illumination, viability of RGCs ranged from 97.5±16.4% of controls with minimum BBG and light exposure (0.25 mg/mL BBG and illuminated for 1 minute at 2.5 cm distance) to 53.1±11.3% of controls with maximum BBG and light exposure (0.50 mg/mL and illuminated for 15 minutes at 1 cm distance; P < 0.01). With medium-intensity illumination, RGCs showed better viability, ranging from 95.1±7.2% of controls with minimum BBG and light exposure to 72.3±12.8% of controls with maximum BBG and light exposure. CellTiter 96^®^ AQueous One assay showed similar results.

**Conclusion:**

RGCs seem to safely tolerate up to 5 minutes of exposure to 0.5 mg/mL BBG under diffuse medium-intensity illumination (990 Fc).

## Introduction

Internal limiting membrane (ILM) peeling improves surgical outcomes in idiopathic macular hole, diabetic macular edema and epiretinal membrane [1, 2]. ILM, a thin transparent membrane, is often difficult to visualize during vitreoretinal surgery and complementary staining with vital dyes, such as indocyanine green (ICG), improves visualization of the ILM intraoperatively and facilitates its safe removal [2, 3]. However, adverse events including retinal pigment epithelial toxicity and visual field changes have been documented after ICG-assisted peeling of the ILM [4-8]. Several alternative dyes such as infracyanine green, bromophenol blue (BPB) and brilliant blue green (BBG) have been proposed as ILM staining agents in vitreomacular surgery. Of those, BBG has shown better affinity to stain ILM with no significant *in vitro* or *in vivo* toxicity [9-11].

Light-induced decomposition of vital dyes may cause retinal injury during chromovitrectomy. Phototoxicity occurs due to absorption of photons emitted by the intraoperative light pipe by the dye-stained retina. Phototoxicity of vital dyes, such as BBG, depends on the type of light source, the intensity of illumination, the distance of the light source from the surface of the retina, and the duration of exposure. Retinal ganglion cells (RGCs) are in direct contact with BBG and may be susceptible to damage from phototoxicity, chemotoxicity or a combination of both. Effects of ICG on RGCs in the presence of endoillumination have been described [12]. Phototoxic effects of BBG on RGCs, however, have not yet been described or investigated.

In this study, we evaluated the effect of surgically used concentrations of BBG on RGC cells after illumination with metal halide light source (common light source used in vitrectomy) at varying distances to identify safety parameters of dye concentration and level of illumination for optimal intraoperative use. RGCs not exposed to BBG, but illuminated by the light source, served as controls.

## Methods

### RGC-5 culture

RGC-5 (Dr. Agarwal, University of Texas, TX) were cultured under standard conditions using Dulbecco’s modified Eagle’s medium (DMEM, L-glutamine, 110 mg/L sodium pyruvate) supplemented with 10% fetal bovine serum (Invitrogen Corp., Carlsbad, CA, USA) and 100 U/mL of penicillin and 100 μg/mL of streptomycin (Invitrogen Corp.). Cells were cultured in 75 cm^2^ filter-capped flasks and maintained in an incubator containing 95% air and 5% CO_2_ at 37 °C.

### Preparation of BBG

BBG (Sigma-Aldrich, St. Louis, MO, USA) was dissolved in Hank’s Balanced Saline Solution (HBSS, Gibco BRL, Invitrogen Corp.) to obtain the concentrations of 0.25 and 0.5 mg/mL (that are described for intraocular use [11]). BBG application was performed in the dark and the culture dishes were protected from light using aluminum foil prior to exposure for 1, 5 and 15 min time intervals at two different dye concentrations.

### Light illumination

**Figure 1:**
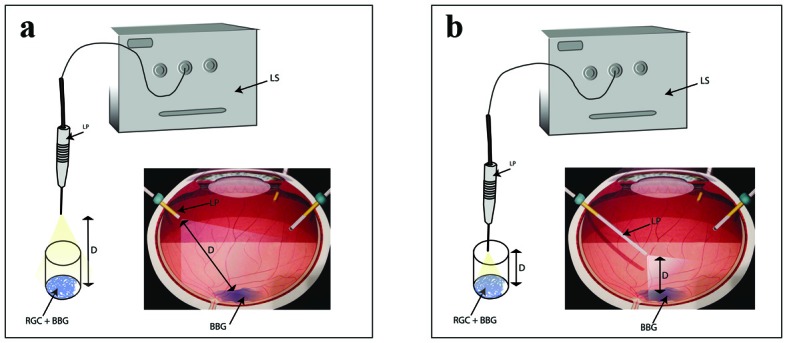
Schematic representation explaining the exposure of RGCs to BBG during intraocular surgery as well as cell culture setup. (a) Diffuse light illumination. (b) Focal illumination. LS: light source; LP: light pipe; D: distance; RGC: retinal ganglion cell; BBG: brilliant blue green.

We used metal halide focal light source without filters to execute the experiments (D.O.R.C. Hexon Halide Light Source, The Netherlands). The straight unfiltered, focal standard 20-gauge fiberoptic endoillumination probe (Synergetics Inc., O’Fallon, MO, USA) was placed at the distances of 1 and 2.5 cm over the cell culture dish to obtain a uniform illumination. The distance between the dish and light source was chosen based on the approximate evaluation of the working distance of light and the retinal surface during membrane peeling (Fig. 1). During exposure to BBG each well/dish was individually illuminated with the metal halide light source at designated time points. After the exposure and/or illumination, cells were rinsed thrice with HBSS (to ensure dye color did not interfere with the results) and evaluated for cytotoxicity. Three series of three experiments at each time point were performed. Experiments without BBG in presence of light illumination served as controls.

### Standardization of light intensity

**Figure 2:**
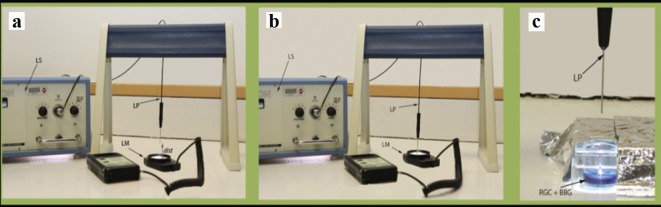
Experimental illustration. (a) Measurement of metal halide light illumination using a light meter at a certain distance from the light source. (b) Measurement of metal halide light illumination using a light meter directly from the light source. (c) Exposure of cultured RGCs to metal halide illumination. LS: light source; LP: light pipe; LM: light meter; dist: distance; RGC: retinal ganglion cell; BBG: brilliant blue green.

We evaluated the intensity of light at the tip of light pipe (source) using a light meter with a 0 - 2,000 Fc range (foot candles; Fig. 2; Extech Instruments Corporation, Waltham, MA, USA). We used high and medium illumination based on the fixed settings on microsurgical system. The illumination levels at high and medium illuminations at the source were at the maximal range and 1,000 Fc, respectively. At high illumination at the surface of the cells, the intensities of light were 2,000 and 915 Fc at the distances of 1 and 2.5 cm, respectively. At the medium illumination level, metal halide light intensities at the cell surface were 990 and 786 Fc at the distances of 1 and 2.5 cm, respectively.

### *In vitro* cytotoxicity analysis

Ten thousand cells/well were seeded in 24-well culture plates and maintained to reach 60-80% confluence (48 - 72 h) prior to the exposure of BBG. After treatment, cell viability was measured using a WST-1 (4-[3-(4 iodophenyl)-2-(4-nitrophenyl)-2H-5-tetrazolio]-1,3-benzene disulfonate), a colorimetric assay (Roche, Mannheim, Germany). This assay is based on the cleavage of tetrazolium salts to formazan by mitochondrial dehydrogenases in viable cells. Cells were washed three times after the exposure using 0.5 mL HBSS, followed by incubation at 37 °C with WST-1. After 2 h, the absorbance was read using a microplate reader (BioTek Synergy HT, Winooski, VT, USA) at 440 nm with a reference wavelength at 630 nm. Results were normalized against controls, and presented as percentage of cell viability.

### CellTiter 96^®^ AQueous One solution cell proliferation assay

After treatment, cell viability was measured using CellTiter 96^®^ AQueous One, a colorimetric assay (Promega, USA). This assay is based on the cleavage of tetrazoliumsalts [3-(4,5-dimethyl-2-yl)-5-(3-carboxymethoxyphenyl)-2-(4-sulfophenyl)-2H-tetrazolium, inner salt; MTS] and an electron coupling reagent (phenazineethosulfate; PES). PES with MTS forms a stable solution. The MTS tetrazolium compound (Owen’s reagent) is bio-reduced by metabolically active cells into a colored formazan product (mediated by dehydrogenase enzymes by NADPH/NADH) that is soluble in tissue culture medium. The absorbance was read at 490 nm using a microplate reader (BioTek Synergy) followed by the incubation with CellTiter 96^®^ AQueous One solution in culture medium. Results were normalized against controls with illumination and presented as percentage of cell viability.

### Statistical analysis

Data were statistically analyzed using GraphPadInstat software (GraphPad Instat3, LaJolla, CA, USA). Statistical significance of differences between groups was compared using ANOVA with *post hoc* Tukey’s test. Statistical significance was accepted for P values of less than 0.05.

## Results

### Cytotoxic effects of distance, duration and intensity of illumination on BBG soaked RGC (WST-1)

#### At 1 cm distance of illumination

Under higher illumination of 2,000 Fc at 0.25 mg/mL, cell viabilities after 1, 5 and 15 min of exposure were 89.8±7.4%, 79.6±4.9% and 56.7±4.0%, respectively. At 0.5 mg/mL concentration, cell viabilities at similar time points were 79±13.1%, 66.7±13.6% and 53.1±11.3% respectively (P = 0.0002) (Table 1).

**Table 1:** Viability of RGCs After Illumination at 1 cm and 2.5 cm Distance With Higher and Medium Illumination of Metal Halide Light Source Using the WST-1 Assay

Distance of illumination (cm)	Concentration of dye (mg/mL)	Higher illumination cell viability ± SD (%)			Medium illumination cell viability ± SD (%)		
		1 min	5 min	15 min	1 min	5 min	15 min
1	0.25	89.8 ± 7.4	79.6 ± 4.9	56.7 ± 4.0	99.1 ± 14.5	94.4 ± 18.8	73.7 ± 16.0
	0.5	79 ± 13.1	66.7 ± 13.6	53.1 ± 11.3	96.5 ± 8.5	84.5 ± 11.0	72.3 ± 12.8
2.5	0.25	97.5 ± 16.4	96.7 ± 15.2	92.4 ± 15.2	95.1 ± 7.2	93.8 ± 7.2	93.0 ± 1.8
	0.5	98.9 ± 12.6	94.8 ± 12.4	82.7 ± 15.7	94.5 ± 3.9	94.0 ± 2.9	87.0 ± 7.0

**Figure 3:**
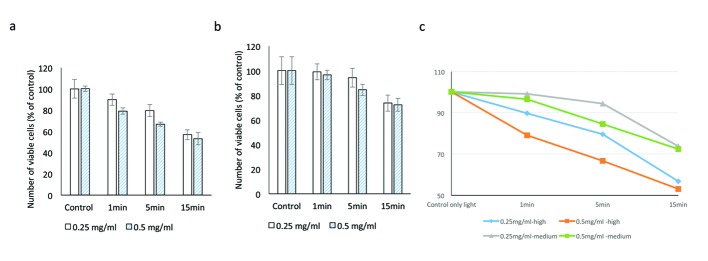
Evaluating the cytotoxic effect of brilliant blue green at 1 cm metal halide distance illumination on retinal ganglion cells using WST-1 assay at high (a) and medium illumination (b). X-axis represents the time dependent exposure in minutes; Y-axis represents number of viable cells expressed as percent of control (N = 4); (c) represents the trend line.

At medium illumination (990 Fc) at 0.25 mg/mL, cell viabilities after 1, 5 and 15 min of exposure were 99.1±14.5%, 94.4±18.8% and 73.7±16.0% respectively. At 0.5 mg/mL, cell viabilities at similar time points were 96.5±8.5%, 84.5±11.0% and 72.3±12.8% respectively (P = 0.001; Fig. 3).

#### At 2.5 cm distance of illumination

With high illumination (915 Fc) and 0.25 mg/mL of BBG, cell viabilities at successive exposure times were 97.5±16.4% (1 min), 96.7±15.2% (5 min), and 92.4±15.2% (15 min). At 0.5 mg/mL concentration, observed cell viabilities were 98.9±12.6% (1 min), 94.8±12.4% (5 min), and 82.7±15.7% (15 min).

**Figure 4:**
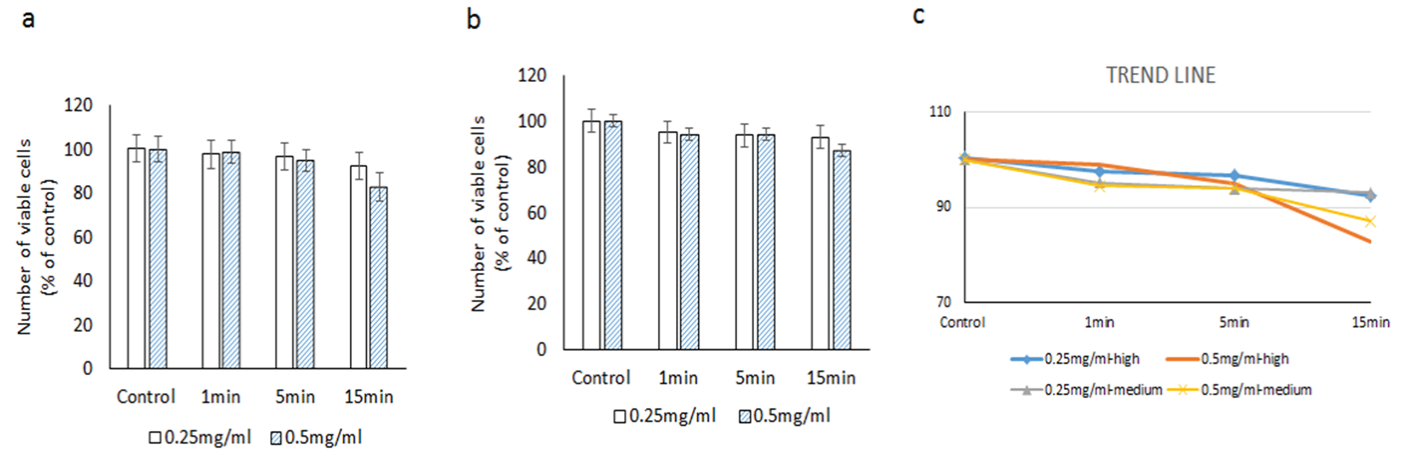
Evaluating the cytotoxic effect of brilliant blue green at 2.5 cm metal halide distance illumination on retinal ganglion cells using WST-1 assay at high (a) and medium illumination (b). X-axis represents the time dependent exposure in minutes; Y-axis represents number of viable cells expressed as percent of control (N = 4); (c) represents the trend line.

At medium intensity illumination (786 Fc) at 0.25 mg/mL concentration, cell viabilities after 1, 5 and 15 min of exposure were 95.1±7.2%, 93.8±7.2% and 93.0±1.8% respectively. At 0.5 mg/mL concentration, cell viabilities at similar time points were 94.5±3.9%, 94.0±2.9% and 87.0±7.0%, respectively (Fig. 4).

### CellTiter 96^®^ AQueous One solution cell proliferation assay

#### At 1 cm distance of illumination

**Figure 5:**
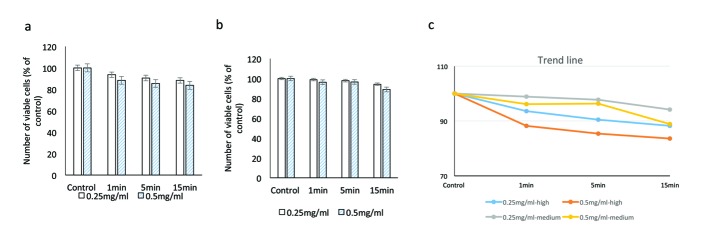
Evaluating the cytotoxic effect of brilliant blue green at 1 cm metal halide distance illumination on retinal ganglion cells using CellTiter 96^®^ AQueous One proliferation assay at high (a) and medium illumination (b). X-axis represents the time dependent exposure in minutes; Y-axis represents number of viable cells expressed as percent of control (N = 4); (c) represents the trend line.

At higher illumination (2,000 Fc) at 0.25 mg/mL concentration, cell viabilities after 1, 5 and 15 min of exposure were 93.5±5.3%, 90.4±3.1% and 88.2±6.7% respectively. At 0.5 mg/mL of BBG, cell viabilities at similar time points were 88.2±5.5%, after 5 min 85.3±7.9% and 83.6±4.1% after 15 min exposure, respectively (Fig. 5) (Table 2).

**Table 2:** Viability of RGCs After Illumination at 1 cm and 2.5 cm Distance With Higher and Medium Illumination of Metal Halide Light Source Using the CellTiter 96^®^ AQueous One Assay

Distance of illumination (cm)	Concentration of dye (mg/mL)	Higher illumination cell viability ± SD (%)			Medium illumination cell viability ± SD (%)		
		1 min	5 min	15 min	1 min	5 min	15 min
1	0.25	93.5 ± 5.3	90.4 ± 3.1	88.2 ± 6.7	98.9 ± 6.5	97.8 ± 7.9	94.2 ± 4.8
	0.5	88.2 ± 5.5	85.3 ± 7.9	83.6 ± 4.1	96.2 ± 8.1	96.4 ± 5.9	88.9 ± 7.1
2.5	0.25	95.6 ± 1.8	91.2 ± 3.2	84 ± 2.3	97.2 ± 7.6	93.7 ± 8.6	91.4 ± 7.9
	0.5	93.3 ± 1.3	83.2 ± 2.0	83.1 ± 2.8	96.3 ± 8.5	91.6 ± 6.9	88.2 ± 7.8

Under medium illumination (990 Fc) at 0.25 mg/mL concentration, cell viabilities after 1, 5 and 15 min of exposure were 98.9±6.5%, 97.8±7.9% and 94.2±4.8% respectively. At 0.5 mg/mL, cell viabilities after 1, 5 and 15 min of exposure were 96.2±8.1%, 96.4±5.9% and 88.9±7.1%, respectively (Fig. 5).

#### At 2.5 cm distance of illumination

Under high illumination (915 Fc) at 0.25 mg/mL concentration, cell viabilities after 1, 5 and 15 min of exposure were 95.6±1.8%, 91.2±3.2%, and 84.0±2.3%, respectively. At 0.5 mg/mL concentration, cell viabilities at similar concentration were 93.3±1.3%, 83.2±2.0%, and 83.1±2.8%, respectively.

**Figure 6:**
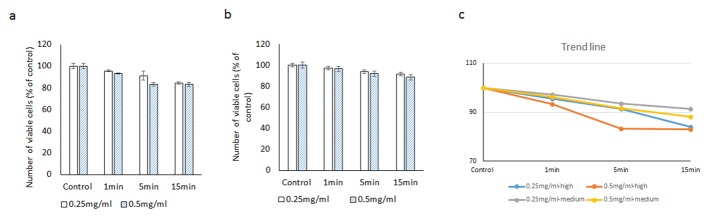
Evaluating the cytotoxic effect of brilliant blue green at 2.5 cm metal halide distance illumination on retinal ganglion cells using CellTiter 96^®^ AQueous One proliferation assay at high (a) and medium illumination (b). X-axis represents the time dependent exposure in minutes; Y-axis represents number of viable cells expressed as percent of control (N = 4); (c) represents the trend line.

Under medium intensity illumination (786 Fc) at 0.25 mg/mL concentration, cell viabilities after 1, 5 and 15 min of exposure were 97.2±7.6%, 93.7±8.6% and 91.4±7.9%, respectively. At 0.5 mg/mL concentration, cell viabilities at similar time points were 96.3±8.5%, 91.6±6.9% and 88.2±7.8%, respectively (Fig. 6).

## Discussion

The ILM plays a significant role in the development of vitreomacular interface diseases, such as idiopathic macular hole (MH), diabetic macular edema (DME) and epiretinal membrane (ERM). In the absence of any randomized controlled trials, peeling of ILM has been a source of debate among vitreoretinal surgeons. Currently many surgeons believe that successful surgical management of diseases of vitreoretinal interface depends on peeling of the ILM, which is thought to be a pivotal source of tangential traction on the retina. ILM peeling may also improve long-term visual outcomes, as it removes the scaffold for possible formation of future ERM, and may prevent re-opening of the macular hole in cases of macular hole surgery [1, 13, 14].

Since the ILM is a transparent thin layer and is barely visible under the microscope, its peeling is a technically challenging procedure. Better visualization of ILM is especially important to minimize the damage to the underlying neuroretinal tissue. Hence, vital dyes are now widely used to stain the ILM and assist in its peeling. Staining the ILM increases the surgical success rates and decreases the incidence of mechanical trauma to the macula [15].

ICG has been used as the most popular dye to selectively stain ILM [16]. The growing experimental evidence, however, has shown that ICG might not be as safe as it was originally thought. *In vitro* studies have shown toxic effects of ICG on RGCs as well as retinal pigment epithelial cells [7, 8]. Clinically, postoperative unfavorable visual acuity outcomes, peripheral visual field defects, RPE atrophy, as well as optic atrophy have been attributed to the use of ICG [4-6, 10, 17]. In addition, illumination may also play a role in ICG-mediated retinal toxicity. When rat retinas were exposed to ICG with and without illumination, retinal damage was significantly more in the presence of illumination [18]. Similarly, when human RPE and rat neurosensory retinal cells were exposed to different concentrations of ICG with and without light exposure, the presence and duration of light exposure was found to be a significant factor in mediating cell toxicity [19].

Consequently, alternative vital dyes have been evaluated to facilitate ILM staining and its removal [9-11, 20]. An ideal replacement to ICG should have maximum ILM staining ability and minimum toxicity to RPE and retinal cells. Among the numerous dyes tested, infracyanine green (IfCG), BBG, and BPB show the highest affinity for ILM [21-23]. Of those, BBG has shown the best affinity for staining the ILM (comparable to ICG) with no significant *in vivo* toxicity [10].

BBG was first introduced as a capsular staining agent for continuous curvilinear capsulorrhexis and ILM staining during vitrectomy for MH repair and ERM removal [22, 24, 25]. Animal studies of subretinal and intravitreal injections of BBG have shown favorable safety profile compared to ICG [26, 27]. When comparing the efficacy and safety of BBG, trypan blue (TB), and ICG in assisting ILM peeling during MH surgery, BBG was similar to ICG in its ease of ILM staining, but showed better final visual outcomes 6 months postoperatively [10].

Despite the improved clinical outcomes with the use of BBG in chromovitrectomy, *in vitro* side effects of BBG have been reported as well. Yuen et al found BBG (among other dyes) to be toxic to human RPE and murine retinal ganglion/Muller cells at higher concentrations [9]. Similarly, Balaiya et al showed that BBG induced necrosis of retinal pigment epithelial cells (ARPE-19) and RGC-5 after more than 5 min of exposure time [11].

RGCs and their axons in the nerve fiber layer form the innermost cellular layers of retina, making them directly exposed to BBG and the stained ILM during chromovitrectomy. BBG has its highest concentration directly adjacent to these layers since it likely gets progressively diluted as it passes through the more outer layers of the retina. Direct exposure of RGCs to BBG may potentially lead to visual field deficits after BBG-assisted chromovitrectomy. Therefore, investigating the safety parameters of using BBG (concentration, during of exposure, amount of illumination) in the context of its side effects on RGCs is important in achieving safer (and more effective) chromovitrectomy using this dye.

In this study, we evaluated the effect of two commonly used concentrations of BBG on RGCs while being illuminated with metal halide light at varying intensities and illumination distances in order to identify optimal safety parameters for the intraoperative use of BBG. RGCs not exposed to BBG (but exposed to metal halide illumination) served as controls. The study was designed to mimic the clinical settings of chromovitrectomy with ILM peeling. We chose metal halide endoillumination as a commonly used light source by many vitreoretinal surgeons. The two intensity levels of this light source (medium and high) and the distances between the tip of the light pipe and the RGCs (1 and 2.5 cm) were also chosen to mimic surgical conditions during vitrectomy (Fig. 2). We chose to investigate the viability of RGCs for the study because they form the innermost cellular layer of the retina, and hence are directly exposed to BBG. The thickness of the RGC layer correlates with central and peripheral vision as demonstrated in glaucoma patients [28]. Therefore, protecting RGCs during any kind of intraocular surgery is essential for the success of the surgery.

We have previously demonstrated that BBG causes necrosis of RPE cells when the exposure time is beyond 5 min [11]. Similarly, BBG was found to cause RPE toxicity in medium (30 min) and long (2 - 72 h), but not short (3 min) exposure times [9]. Since short to medium exposure times are more surgically relevant, we used 1, 5 and 15 min as exposure times in the present study. We demonstrated that longer RGC exposure time to BBG generally leads to decreased cell viability regardless of BBG concentration and illumination conditions. Nonetheless, the exposure time had minimum effect of cell viability when diffuse illumination (2.5 cm) was used and BBG was used at the low (0.25 mg/mL) concentration. The exposure time had maximal effect on cell viability when high illumination intensity was used at focal (1 cm) illumination and the cells were exposed to high (0.5 mg/mL) concentration of BBG.

In the presence of endoillumination, the concentration of ICG correlates with its toxicity on RPE cells [29]. Similarly, successively lower concentrations of BBG were found to be associated with higher viability of cultured RPE cells [9]. Both of our cell viability assays showed that regardless of the BBG exposure time and illumination intensity, RGCs that were exposed to the higher concentration of BBG (0.5 mg/mL) had lower cell viability compared to those that were exposed to lower concentration (0.25 mg/mL). This effect was particularly prominent with the focal (1 cm) compared to the diffuse (2.5 cm) illumination.

The type and intensity of the light source used in chromovitrectomy can potentially influence the cellular toxicity of vital dyes. The intensity of the illuminating light source as a variable in chromovitrectomy, however, has not been investigated before. In our study we used a light meter to measure the light intensity (expressed as foot candles to indicate the lumens of light per unit area) of the metal halide light at the illumination source and, more importantly, at the cells surface (Fig. 1a, b). Both cell viability assays showed that with the focal (1 cm) illumination distance, cells illuminated with medium light intensity (990 Fc at the source) had significantly better viability compared to those that were illuminated with the higher intensity illumination (2,000 Fc or above at the source). Interestingly, this difference was much less prominent when cells were exposed to diffuse (2.5 cm) illumination. In other words, regardless of BBG concentration and exposure time, diffuse illumination leads to less RGC phototoxicity even when higher intensity illumination is used. Our results show that BBG has cytotoxic effects on cultured RGCs especially when used with longer exposure time, higher concentration, higher illumination intensity, and shorter illumination distance. The maximal cytotoxicity was observed when RGCs were exposed for 15 min to 0.5 mg/mL BBG and focally illuminated with a high intensity (2,000 Fc) light source.

In conclusion, the maximal tolerated limit of BBG and light exposure of RGCs, which can be used to guide the safe clinical application of BBG in chromovitrectomy while maximizing its staining properties, seems to be when the cells are exposed for up to 5 min to 0.5 mg/mL BBG and diffusely illuminated (at 2.5 cm) with medium (990 Fc) light intensity.
